# The adult *Drosophila* testis lacks a mechanism to replenish missing niche cells

**DOI:** 10.1242/dev.201148

**Published:** 2023-01-20

**Authors:** Phylis Hétié, Margaret de Cuevas, Erika L. Matunis

**Affiliations:** Department of Cell Biology, Johns Hopkins University School of Medicine, 725 N. Wolfe Street, Baltimore, MD 21205, USA

**Keywords:** Stem cell, Niche, Spermatogenesis, Cell ablation, GeneSwitch

## Abstract

The adult *Drosophila* testis contains a well-defined niche created by a cluster of hub cells, which secrete signals that maintain adjacent germline stem cells and somatic cyst stem cells (CySCs). Hub cells are normally quiescent in adult flies but can exit quiescence, delaminate from the hub and convert into CySCs after ablation of all CySCs. The opposite event, CySC conversion into hub cells, was proposed to occur under physiological conditions, but the frequency of this event is debated. Here, to probe further the question of whether or not hub cells can be regenerated, we developed methods to genetically ablate some or all hub cells. Surprisingly, when flies were allowed to recover from ablation, the missing hub cells were not replaced. Hub cells did not exit quiescence after partial ablation of hub cells, and labeled cells from outside the hub did not enter the hub during or after ablation. Despite its ability to exit quiescence in response to CySC ablation, we conclude that the hub in the adult *Drosophila* testis does not have a mechanism to replenish missing hub cells.

## Teaser

**Highlighted Article:** Non-dividing niche cells in the adult *Drosophila* testis, which are able to divide to replace missing stem cells, are unable to replace themselves.

## INTRODUCTION

**Fig. 1.**
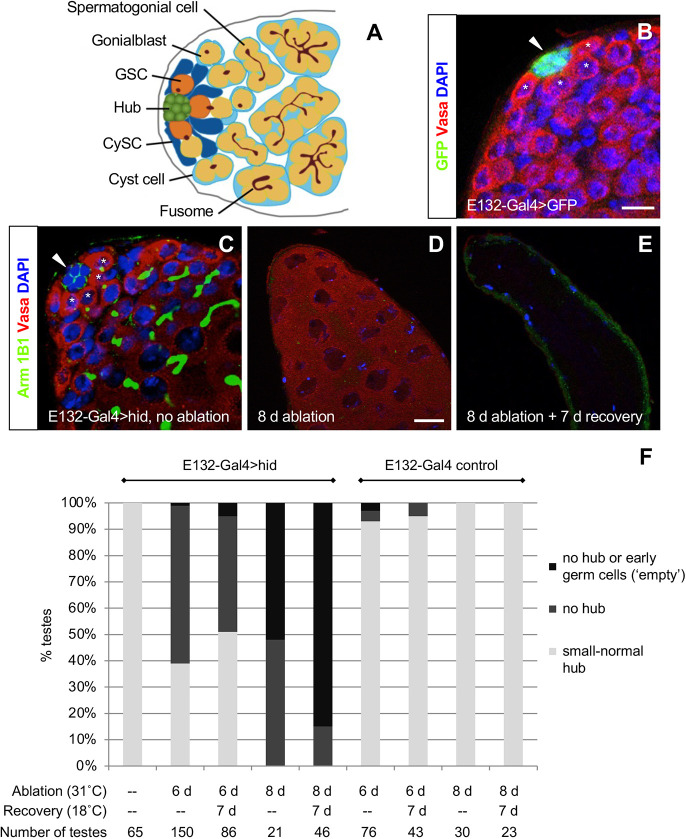
**The hub is not replaced after complete genetic ablation of hub cells in adult testes.** (A) Diagram of the *Drosophila* testis apex. Germline stem cells (GSCs, orange) and somatic cyst stem cells (CySCs, dark blue) adhere to quiescent somatic hub cells (green). GSCs produce gonialblasts (yellow), which give rise to clusters of spermatogonia. CySCs produce cyst cells (light blue), which envelop differentiating germ cells. The fusome (brown) is round in GSCs and gonialblasts, and elongated or branched in spermatogonia. (B) Single confocal section through the testis apex from an *E132-Gal4>GFP* fly stained with anti-GFP, anti-Vasa (germ cells), and DAPI (DNA). *E132-Gal4* drives robust expression of GFP in hub cells (arrowhead). Asterisks mark GSCs. (C-E) Testes from *E132-Gal4/Gal80^ts^>hid* flies stained with anti-Vasa, anti-Arm (hub cell membranes), 1B1 (fusomes) and DAPI. Before *hid* induction (C), testes appear wild type and contain a hub (arrowhead) surrounded by GSCs (asterisks) with round fusomes. After 8 days of *hid* induction (D), all testes lack hub cells and most lack early germ cells. (The testis apex shown here contains only older spermatocytes, which stain less brightly with anti-Vasa.) After 7 days of recovery (E), no hub cells are regained and most testes appear ‘empty’. Scale bars: 10 μm (in B, for B,C); 20 μm (in D, for D,E). (F) The percentage of testes with or without hubs in experimental *(E132-Gal4/Gal80^ts^>hid)* or control *(E132-Gal4/Gal80^ts^)* flies. d, days.

Tissue homeostasis and repair depend on adult stem cells, which are maintained and regulated by local microenvironments called niches. The *Drosophila* testis contains a single niche composed of a cluster of 10-15 hub cells surrounded by two types of stem cells, germline stem cells (GSCs) and somatic cyst stem cells (CySCs) (Fig. 1A) (reviewed by Greenspan et al., 2015). GSCs produce spermatogonia and CySCs produce cyst cells, which encase spermatogonia and are essential for their differentiation into sperm. The hub secretes signals that are required to maintain adjacent stem cells and also serves as the organizing center of the niche; stem cells adhere to the hub and divide perpendicularly to it, ensuring that one daughter remains in the niche, whereas the other is displaced and can differentiate (Hardy et al., 1979). GSCs, CySCs and hub cells are well-characterized and easy to distinguish, which makes the *Drosophila* testis an ideal model for understanding niche-stem cell interactions.

Hub cells and CySCs are specified early in embryogenesis from a common pool of somatic gonadal precursor cells, but, once specified, the two cell types adopt opposing fates: CySCs continue to proliferate while hub cells become quiescent (DiNardo et al., 2011; Le Bras and Van Doren, 2006; Zoller and Schulz, 2012). Although they remain quiescent throughout the lifetime of the fly, hub cells retain the capacity to exit quiescence and generate new CySCs: genetic ablation of all CySCs causes hub cells to proliferate, leave the hub and convert into functional CySCs (Hétié et al., 2014). Hub cells do not proliferate or convert into CySCs after ablation of only some CySCs, suggesting that signals from remaining CySCs block these processes in hub cells (Hétié et al., 2014; Herrera et al., 2021; Greenspan et al., 2022). However, hub cells can be induced to proliferate and convert to CySCs even in testes that have lost no CySCs by forced expression of Cyclin D and Cyclin-dependent kinase 4 (Hétié et al., 2014), by knockdown of the cell cycle inhibitor Retinoblastoma-family protein (Greenspan and Matunis, 2018), or by activation of the EGFR/MAPK pathway (Greenspan et al., 2022) specifically in hub cells. Hub cells can also be induced to convert to CySCs without replenishment of lost hub cells, resulting in the complete loss of the hub over time, for example upon knockdown of the transcription factor Escargot (Voog et al., 2014) or upon activation of the Activin pathway (Herrera et al., 2021). In these cases, it is likely that hub cells change fate and exit the hub before losing quiescence, although this has not been directly tested. By contrast, whether or not CySCs can give rise to new hub cells is controversial. Hub cells derived from CySCs were reported in one study (Voog et al., 2008), but another study, which used the same genetic tools, found no evidence for this (DiNardo et al., 2011). Therefore, whether or not the testis has the capacity to replace lost hub cells – by proliferation of CySCs and their conversion to hub cells or by proliferation of remaining hub cells – was not known.

## RESULTS AND DISCUSSION

### Hub cells are not replenished after complete ablation of the hub

We first established conditions for genetically ablating all hub cells. We conditionally expressed the pro-apoptotic gene *head involution defective* (*hid*) in hub cells in adult testes using the temperature-inducible Gal4-UAS-Gal80^ts^ system (McGuire et al., 2003), the hub-specific driver E132-Gal4 (Fig. 1B) and a *UAS-hid^Ala5^* transgene (Bergmann et al., 1998, 2002). Before induction (18°C), *E132-Gal4/Gal80^ts^>hid* testes were phenotypically wild type (Fig. 1C,F). By contrast, after 8 days of *hid* induction (31°C), all hub cells in all testes were ablated, and early germ cells and cyst lineage cells were also no longer present in most testes (Fig. 1D,F). When flies without hub cells were allowed to recover for 7 days (18°C), all testes remained without a hub. By this time, most testes also lacked spermatocytes and looked ‘empty’ (Fig. 1E,F). We conclude that genetic ablation of all hub cells in adult testes causes an irreversible loss of the hub and that GSCs and CySCs are lost as a consequence of hub ablation. Because signals from hub cells are required for stem cell maintenance (reviewed by Greenspan et al., 2015; Zoller and Schulz, 2012), the loss of GSCs and CySCs upon complete ablation of the hub was expected. GSCs and CySCs are also lost as a consequence of hub cell death after knockdown in hub cells of the anti-apoptotic gene *headcase* (Resende et al., 2013) or the miRNA processing factor *Dicer-1* (Volin et al., 2018).

Having established a tool to ablate all hub cells, we then investigated whether hub cells can be regenerated after some, but not all, of the hub cells in the testis are ablated, referred to here as ‘partial hub ablation’, by inducing *hid* expression in *E132-Gal4/Gal80^ts^>hid* flies for 6 days (instead of 8 days). The extent of hub ablation varied widely: 40% of testes had a normal to small-sized hub and 60% had no hub (*n*=149 testes; Fig. 1F). After 7 days of recovery, the percentage of testes with or without a hub remained about the same: 51% had a normal to small-sized hub and 49% had no hub (*n*=86 testes, *P*>0.05, Fisher's exact test; Fig. 1F). These results are consistent with the idea that lost hub cells are not being replaced, but the wide variation in hub size (from no hub to normal) before and after recovery made it difficult to analyze the data.

### The GeneSwitch system can be used to ablate hub cells

**Fig. 2.**
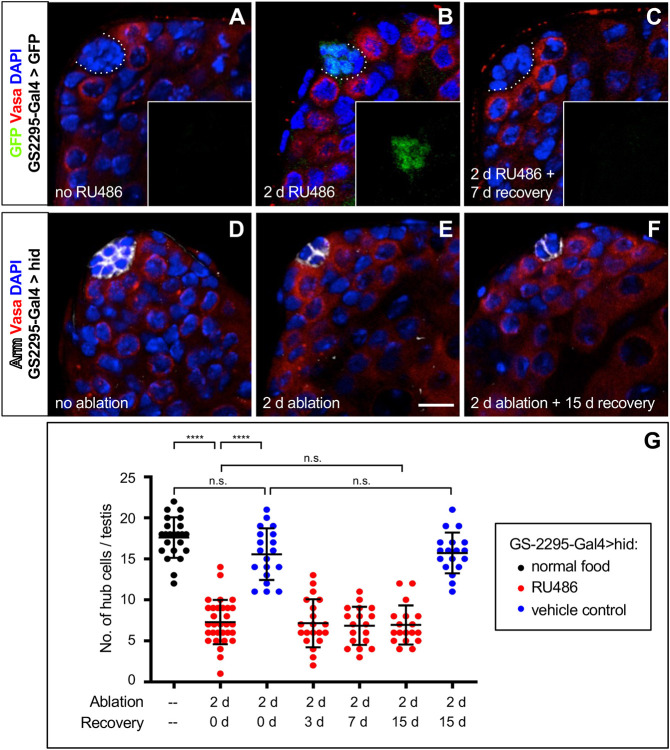
**Hub cells are not replaced after partial ablation of the hub in adult testes.** (A-C) Confocal images of the testis apex from *GS-2295-Gal4>GFP* flies stained with anti-GFP, anti-Vasa and DAPI. Insets show the green channel (GFP) alone. Without RU486 (A), GFP is not detectable. After 2 days on RU486 (B), GFP is detectable in the hub. After recovery (C), GFP is no longer detectable. (D,E) Confocal images of the testis apex from *GS-2295-Gal4>hid* flies stained with anti-Vasa, anti-Arm and DAPI. Without RU486 (D), the hub is a normal size. After 2 days on RU486 (E), the hub is smaller. After recovery (F), the hub remains small. Scale bar: 10 μm (in E, for all panels). (G) The number of hub cells per testis before and after hub cell ablation in *GS-2295-Gal4>hid* flies. Black bars indicate the mean±s.d. The number of hub cells per testis decreases significantly after ablation and remains low. *n*=18-31 testes for all time points. ****P<0.0001; n.s., not significant (*P*>0.05). d, days.

Searching for a driver that could induce a more uniform partial ablation of the hub, we screened a collection of GeneSwitch-Gal4 (GS-Gal4) lines (Nicholson et al., 2008) for expression in hub cells. GS-Gal4 is a fusion protein composed of the DNA-binding domain of Gal4 and the ligand-binding domain of the progesterone receptor (Osterwalder et al., 2001; Roman et al., 2001). Inactive on its own, it becomes active in the presence of the synthetic steroid RU486 (mifepristone). We identified one line, *GS-2295-Gal4*, that drives expression of a *UAS-GFP* transgene in hub cells. We raised *GS-2295-Gal4>GFP* flies to adulthood on normal food (without RU486) and saw no GFP expression above background levels in the testis apex (Fig. 2A; *n*=17 testes). We then transferred flies to vials containing 1 mg/ml RU486 in apple juice; after 2 days, GFP was expressed specifically in the hub in all testes (Fig. 2B; *n*=60 testes). To determine whether hub-specific RU486-induced gene expression is reversible, we returned RU486-fed flies to normal food to recover. After 3 days, GFP expression was still apparent in some testes (*n*=40 testes), but by 7 days of recovery, GFP expression was no longer visible in the hub in any testis (Fig. 2C; *n*=37 testes). We conclude that *GS-2295-Gal4* can drive the reversible expression of *UAS* transgenes specifically in the hub. Because the number of hub cells per testis in *GS-2295-Gal4>GFP* flies is about the same with or without RU486 feeding (Fig. S1), we also conclude that RU486 by itself does not affect hub cell number.

We next investigated whether we could use *GS-2295-Gal4* to ablate hub cells in adult testes by conditionally expressing *hid*. Before *hid* induction (on normal food), testes from *GS-2295-Gal4>hid* flies resembled wild-type testes and had a phenotypically wild-type number of hub cells (Fig. 2D,G). We then transferred *GS-2295-Gal4>hid* flies to vials containing either RU486 (to induce *hid*) or vehicle only (uninduced control) in apple juice. Uninduced control flies retained a phenotypically wild-type number of hub cells (about 16 cells; Fig. 2G). By contrast, after two days of *hid* induction (on RU486), the average number of hub cells per testis (about seven; Fig. 2E,G) was significantly lower than in uninduced control flies. Importantly, almost all testes retained at least a few hub cells, and none of the testes completely lacked a hub. These data indicate that the GeneSwitch system can be used to ablate some hub cells in all testes more uniformly than in *E132-Gal4/Gal80^ts^>hid* flies. We therefore used this driver for the rest of our experiments.

**Fig. 3.**
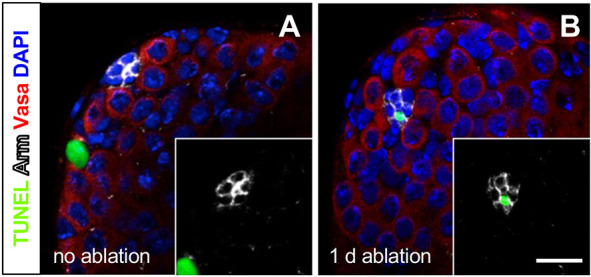
**Hub cells are lost by apoptosis upon genetic ablation of hub cells.** (A,B) Confocal images of the testis apex from *GS-2295-Gal4>hid* flies stained with TUNEL (apoptotic cells), anti-Arm, anti-Vasa and DAPI. Insets show the green and white channels (TUNEL and hub cell membranes) alone. Apoptotic hub cells are rare in uninduced control testes (A), but found in testes after 1 day of *hid* induction in the hub (B). TUNEL-positive cells are common outside the hub in most testes (A). Scale bar: 10 μm (in B, for A,B).

We expected that hub cells were being lost by apoptosis upon induction of *hid* in *GS-2295-Gal4>hid* flies. Using the terminal deoxynucleotidyl transferase dUTP nick end labeling (TUNEL) technique to mark apoptotic cells, we compared the number of testes with apoptotic hub cells in *hid*-induced and uninduced control flies. After 1-2 days of *hid* induction in the hub, 19-20% of testes had at least one TUNEL-positive hub cell, significantly more than control testes, which had 0% TUNEL-positive hub cells at either time point (Fig. 3; Table S1). TUNEL-positive cells were evident outside the hub in both *hid*-induced and control testes, as expected (Hasan et al., 2015; Yacobi-Sharon et al., 2013). We conclude that hub cells are being lost by apoptosis upon induction of *hid* in the hub; however, it is also possible that some hub cells leave the hub before dying. Loss of hub cells by apoptosis, resulting in partial or complete ablation of the hub, also occurs after knockdown of *headcase* or *Dicer-1* in the hub (Resende et al., 2013; Volin et al., 2018).

### Hub cells are not replenished after partial ablation of the hub

To determine whether lost hub cells can be replaced after partial ablation of the hub, we returned *GS-2295-Gal4>hid* flies to standard yeast medium after 2 days on RU486 (or vehicle control) to let them recover. As expected, the average number of hub cells per testis did not change in control flies; they still had a phenotypically wild-type number of hub cells after recovery (Fig. 2G). Surprisingly, there was also no change in the average number of hub cells per testis in RU486-fed flies; after 3, 7 or 15 days of recovery, hubs still had an average of about seven cells (Fig. 2F,G). Early germline and somatic cells were present in the apex of all testes at all time points (Fig. 2F), suggesting that even testes with very few hub cells retained functional stem cells, as expected based on prior work showing that hubs composed of one to three cells can support GSCs and CySCs (Resende et al., 2013). We conclude that hubs remain functional, but missing hub cells are not replaced after partial ablation of the hub. The ability of lost hub cells to be replaced after knockdown of *headcase* or *Dicer-1* in the hub was not assessed (Resende et al., 2013; Volin et al., 2018), but our findings suggest that hub regeneration would not occur in these testes.

We considered the possibility that hub cell number is static after ablation because hub cells continue to die but are replaced by new hub cells at a balanced rate. To ascertain whether hub cells continue to die during recovery, we used TUNEL to mark apoptotic cells in testes from *GS-2295-Gal4>hid* flies at 1-4, 7 and 15 days of recovery. We found TUNEL-positive hub cells in a small percentage of testes recovering from *hid* induction; uninduced control flies had a similar percentage of testes with TUNEL-positive hub cells, as did RU486-fed control flies carrying the *GS-2295-Gal4* driver or *UAS-hid* transgene alone (Table S1). Leakiness of the *UAS-hid* transgene could be contributing to some of this hub cell death. These results suggest that hub cells in *GS-2295-Gal4>hid* flies continue to die after ablation; however, the frequency of hub cell death is significantly lower than during ablation and is indistinguishable from that in control flies. We conclude that hub cell death after ablation, as in control flies, is a rare event that does not cause a significant decrease in hub cell number over the time course of the experiment.

**Fig. 4.**
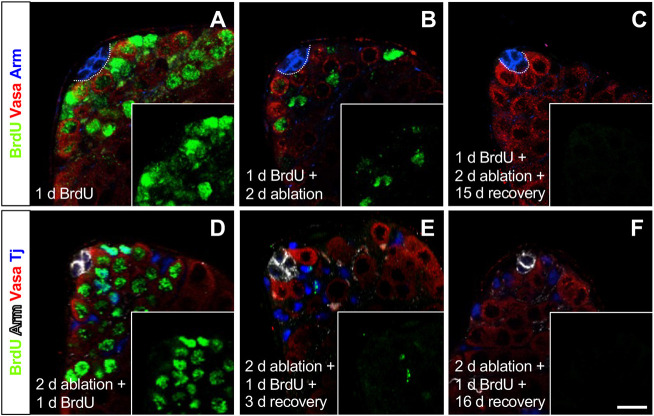
**Hub cells do not proliferate and cycling cells do not enter the hub during or after genetic ablation of hub cells.** (A-C) Confocal images of the testis apex from *GS-2295-Gal4>hid* flies stained with anti-BrdU (cycling cells), anti-Vasa and anti-Arm. Flies were fed BrdU for 1 day to label cycling cells (A), then RU486 for 2 days to induce *hid* expression (B), and allowed to recover on normal food (C). (D-F) Confocal images of the testis apex from *GS-2295-Gal4>hid* flies stained with anti-BrdU, anti-Arm, anti-Vasa and Tj (cyst lineage cells). Flies were fed RU486 for 2 days followed by BrdU for 1 day (D) and then allowed to recover on normal food (E,F). Proliferating germline and somatic cells outside the hub are robustly labeled with BrdU initially (A,D), but we found no BrdU-labeled cells inside the hub in any testis at any time point in either experiment (A-F). Scale bar: 10 μm (in F, for all panels).

If lost hub cells were being replaced by new hub cells, we reasoned that they could arise from cells entering the hub from outside, as previously proposed (Voog et al., 2008), or from the proliferation of remaining cells inside the hub. To establish whether proliferating cells enter the hub during hub cell ablation, we raised *GS-2295-Gal4>hid* flies to adulthood on normal food and transferred them to vials containing the thymidine analog 5-bromo-2′-deoxyuridine (BrdU) in apple juice to label proliferating cells. After 1 day on BrdU, but before *hid* induction, most cells in the testis apex were labeled, including almost all GSCs and CySCs, as expected (Yadlapalli et al., 2011; Hétié et al., 2014; Ma et al., 2014). By contrast, no BrdU-positive hub cells were detected in any testis (*n*=118 testes; Fig. 4A), confirming that hub cells were not actively cycling before ablation. This result also suggests that no cycling cells entered the hub while the flies were on BrdU, consistent with the observation that CySCs rarely generate daughter cells that enter the hub (DiNardo et al., 2011). We then transferred BrdU-fed flies to vials containing RU486 to induce *hid*. After 1 day of BrdU labeling followed by 2 days of *hid* induction, we still observed BrdU-labeled germline and somatic cells outside the hub but none in the hub (*n*=18 testes; Fig. 4B). We considered the possibility that BrdU-labeled cells entered the hub and rapidly proliferated, diluting the label to undetectable amounts; this scenario is unlikely, however, as a previous study showed that BrdU labeling remains detectable in 60% of actively cycling GSCs after a 2-day chase period (Yadlapalli et al., 2011). Therefore, we conclude that no BrdU-labeled cells entered the hub during hub cell ablation. After 1 day of BrdU labeling, 2 days of *hid* induction and 3 days of recovery, BrdU was no longer present in the apex of most testes (*n*=31), and by 15 days of recovery it was no longer present the apex of all testes (*n*=25; Fig. 4C), as expected given that the BrdU-labeled cells were actively cycling and diluting the label (Yadlapalli et al., 2011). We saw no BrdU-labeled cells in the hub at any time point.

To determine whether the remaining hub cells begin cycling or if proliferating cells enter the hub after (rather than during) ablation, we placed adult *GS-2295-Gal4>hid* flies on RU486 for 2 days (to induce *hid*) and then on BrdU for 1 day. Again, most cells in the testis apex were labeled, including almost all GSCs and CySCs, but no hub cells were labeled in any testis (Fig. 4D; *n*=59 testes). This result confirms that no hub cells began cycling and that no BrdU-labeled cells entered the hub from outside in the 24 h after ablation. We then let the *hid*-induced, BrdU-fed flies recover for 3 or 16 days on normal food (Fig. 4E,F; *n*=76 or 51 testes, respectively). No BrdU-labeled cells were apparent in the hub at any time point, supporting the hypothesis that BrdU-labeled cells do not enter the hub during recovery from ablation.

Taken together, our results suggest that flies do not have a mechanism to replace lost hub cells. Both types of stem cells in the testis can be restored after loss – GSCs by dedifferentiation of spermatogonia (Brawley and Matunis, 2004) and CySCs by conversion of hub cells (Hétié et al., 2014) – but lost hub cells are not replaced either by themselves or by conversion of another cell type. Even if remaining hub cells divide or other cells enter the hub on rare occasions, these events are not sufficient to replace missing cells after genetic ablation of hub cells. This finding was unexpected; in many tissues, when large numbers of cells undergo apoptosis, they release signals that stimulate the proliferation of neighboring cells, thereby regenerating the missing cells (reviewed by Bergmann and Steller, 2010; Fuchs and Steller, 2015). Although hub cells can proliferate in response to the complete loss of somatic stem cells, they do not proliferate in unperturbed testes or in testes that have lost some (but not all) CySCs (Hétié et al., 2014). Perhaps the mechanisms that maintain hub cell quiescence in unperturbed testes (Herrera et al., 2021; Greenspan et al., 2022) also prevent the proliferation of remaining hub cells after genetic ablation of hub cells.

## MATERIALS AND METHODS

### Fly stocks and culture

*Drosophila melanogaster* males collected 0-3 days after eclosion were maintained on standard yeast medium unless otherwise noted. All fly stocks came from the Bloomington *Drosophila* Stock Center except *E132-Gal4 (upd-Gal4)*, which was obtained from H. Sun (Academica Sinica, Taipei, Taiwan) and *UAS-hid^Ala5^* (lines 43 and 49) obtained from J. Abrams (UT Southwestern, Dallas, TX, USA). The genotypes of flies used are: *E132-Gal4/Y; UAS-GFP/+* (abbreviated as *E132-Gal4>GFP*); *E132-Gal4/Y; UAS-hid/+; tub-Gal80ts/+* (abbreviated as *E132-Gal4/Gal80ts>hid*); *GS-2295-Gal4/+; UAS-GFP/+* (abbreviated as *GS-2295-Gal4>GFP*); and *UAS-hid/Y; GS-2295-Gal4/+* (abbreviated as *GS-2295-Gal4>hid*).

### RU486 or BrdU feeding

RU486 (50 mg/ml in ethanol) was diluted to 1 mg/ml in apple juice; vehicle control was the same volume of ethanol in apple juice. BrdU was used at 2.5 mM in apple juice. Green food dye (McCormick) was added at 1 µl per 20 µl solution. The mix was pipetted onto filter paper circles in empty fly vials (100-150 µl solution/vial). Flies were cultured at 25°C and transferred daily to fresh vials. Only flies with green guts, which had ingested the drug, were used for experiments.

### Immunofluorescence microscopy

Testes were dissected, fixed and stained as described (Matunis et al., 1997). The following antibodies were used: goat anti-Vasa dC-13 (Santa Cruz Biotechnology, sc-26877; 1:200), rabbit anti-Vasa d-260 (Santa Cruz Biotechnology, sc-30210; 1:400); rabbit anti-GFP (Torrey Pines Biolabs, TP401; 1:10,000); rat anti-BrdU (AbD Serotec, MCA2060; 1:40); mouse anti-Hts, not Hts-PC (Developmental Studies Hybridoma Bank, 1B1; 1:50); mouse anti-Armadillo (Developmental Studies Hybridoma Bank, N2 7A1 Armadillo; 1:50); guinea pig anti-Traffic jam [from D. Godt (University of Toronto, ON, Canada); 1:10,000]; and Alexa Fluor secondary antibodies (Molecular Probes/Invitrogen, A11001, A11006, A11011, A11057, A21105, A21121, A21202, A21206 and A31553; 1:200-1:400). DNA was stained with 4,6-diamidino-2-phenylindole (DAPI, Sigma-Aldrich) at 1 µg/ml. TUNEL (Chemicon International, S7160) was performed as described (Sheng et al., 2009). Testes were imaged with a Zeiss LSM 5 Pascal or LSM 510 Meta laser-scanning microscope.

## Supplementary Material

10.1242/develop.201148_sup1 Supplementary information
