# Supplementary material for: The adult Drosophila testis lacks a mechanism to replenish missing niche cells
Source: Development. 2023 Jan 20;150(2):dev201148. doi: 10.1242/dev.201148 (PMC10110489; doi:10.1242/dev.201148)
Supplement: Supplementary information [file develop-150-201148-s1.pdf]

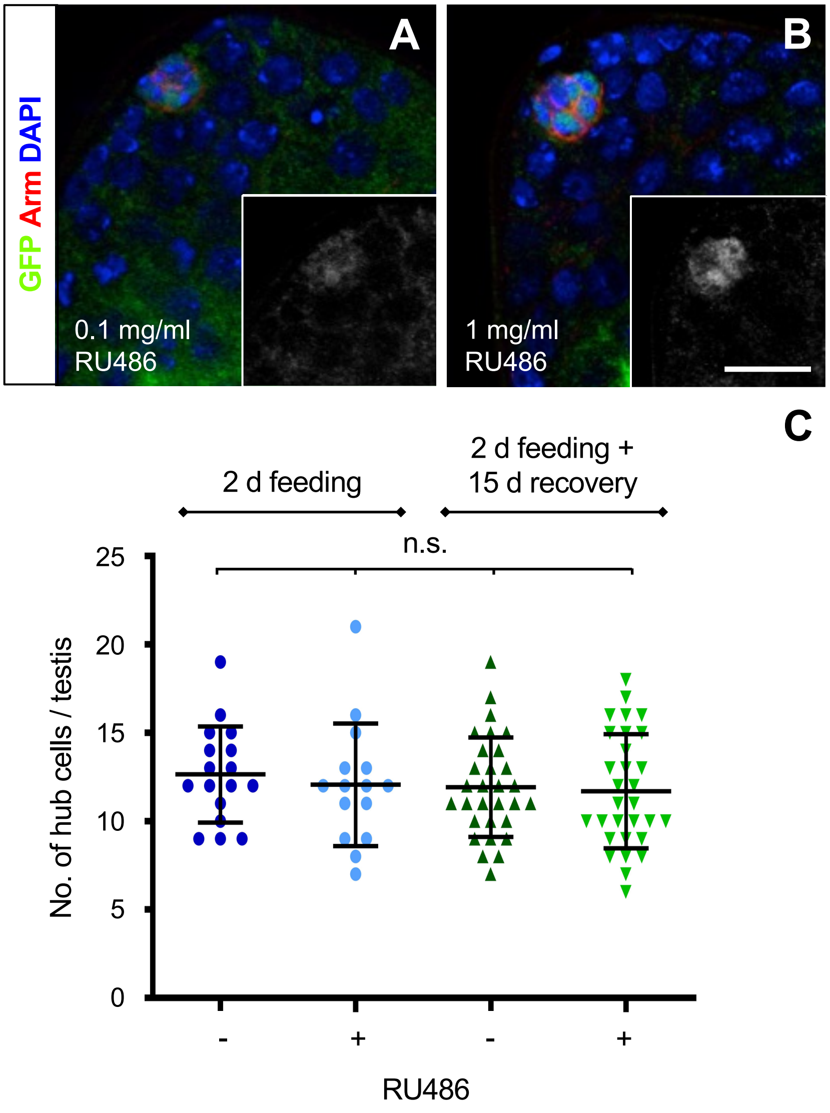

Fig. S1. In *GS-2295-Gal4>GFP* flies, RU486 drives expression of GFP in the hub, but hub cell number is not affected.

(A-B) Confocal images of the adult testis apex from *GS-2295-Gal4>GFP* flies stained with anti-GFP, anti-Arm, and DAPI. Insets show green channel (GFP) alone. Scale bar in B, for A-B, is 10 µm. Flies were fed RU486 at 0.1 mg/ml (A) or 1 mg/ml (B) for 2 days before dissection. GFP is expressed in the hub at higher levels in flies fed the higher dose, suggesting that transgene expression depends on the dose of RU486 as in other tissues (Osterwalder et al., 2001). Expression is not uniform across the hub at either dose. **(C)** Scatter plot showing the number of hub cells per testis in *GS-2295-Gal4>-GFP* flies fed 1 mg/ml RU486 or vehicle control (ethanol) in apple juice for 2 days and allowed to recover on standard yeast medium for 15 days. Black lines indicate the mean ± s.d. No significant difference in hub cell number was observed between experimental and control testes either before or after recovery ($p > 0.05$, one-way ANOVA).

Development • Supplementary information

		Recovery						
**Genotype**	**Feeding**	**0 d**	**1 d**	**2 d**	**3 d**	**4 d**	**7 d**	**15 d**
GS-2295-Gal4>hid	1 d RU486	20% (95)						
GS-2295-Gal4>hid	1 d mock	0% (92)						
GS-2295-Gal4>hid	2 d RU486	19% (100)	4% (97)	2% (89)	2% (199)	1% (159)	1% (174)	1% (135)
GS-2295-Gal4>hid	2 d mock	0% (118)	1% (101)	3% (91)	3% (217)	3% (158)	1% (169)	3% (98)
**GS-2295-Gal4** only	2 d RU486				0% (73)	0% (68)	0% (61)	0% (48)
**UAS-hid** only	2 d RU486				1% (89)	2% (93)	0% (39)	0% (44)

Table S1. Percentage of testes with TUNEL-positive hub cells.

Development • Supplementary information
