# Supplementary material for: The adult Drosophila testis lacks a mechanism to replenish missing niche cells
Source: Development. 2023 Jan 20;150(2):dev201148. doi: 10.1242/dev.201148 (PMC10110489; doi:10.1242/dev.201148)
Supplement: Supplementary file 1 [file develop-150-201148_review_history.pdf]

# The adult *Drosophila* testis lacks a mechanism to replenish missing niche cells

Phylis Hétié, Margaret de Cuevas and Erika L. Matunis
DOI: 10.1242/dev.201148

**Editor**: Cassandra Extavour

**Review timeline**
Original submission: 20 July 2022
Editorial decision: 12 September 2022
First revision received: 1 November 2022
Accepted: 29 November 2022

---

**Original submission**

First decision letter

MS ID#: DEVELOP/2022/201148

MS TITLE: The adult Drosophila testis lacks a mechanism to replenish missing niche cells

AUTHORS: Phylis Hétié, Margaret de Cuevas, and Erika L. Matunis

I have now received all the referees reports on the above manuscript, and have reached a decision. The referees' comments are appended below, or you can access them online: please go to BenchPress and click on the 'Manuscripts with Decisions' queue in the Author Area.

The overall evaluation is positive and we would like to publish a revised manuscript in Development, provided that the referees' comments can be satisfactorily addressed. Please attend to all of the reviewers' comments in your revised manuscript and detail them in your point-by-point response. If you do not agree with any of their criticisms or suggestions explain clearly why this is so. If it would be helpful, you are welcome to contact us to discuss your revision in greater detail. Please send us a point-by-point response indicating your plans for addressing the referee's comments, and we will look over this and provide further guidance.

Reviewer 1

*Advance summary and potential significance to field*

In this work, Hetie et al have followed up on their important discovery that hub cells can exit quiescence to replenish the somatic stem cell population in the testis by addressing a critical and contentious question in the field--can somatic stem cells replenish cells of the hub. By adapting the Geneswitch system for use in the fly testis, the authors conclusively show through a number of elegant experiments that somatic stem cells do not replenish hub cells after partial or complete hub cell loss. It will be of significant interest to the field of stem cell biology to have this question resolved.

*Comments for the author*

While use of Geneswitch as a genetic technique is novel, the results presented in this manuscript are not themselves truly novel. However, this is massively outweighed by the significant impact of

© 2023. Published by The Company of Biologists under the terms of the Creative Commons Attribution License (https://creativecommons.org/licenses/by/4.0/).

these results to the field. The experiments presented are elegant and thoughtful with the results straightforward to interpret. The question of whether somatic stem cells can replace niche cells in the fly testis has been a particularly contentious issue. Experiments conducted by a lab with precisely the appropriate expertise to address this question and with experiments that provide clear-cut results is necessary for the field and as such I think warrants publication in this journal.

A minor question remains as to why control testes experience hub cell loss during recovery following RU486 treatment. However, this in no way impacts the actual results/interpretation of the manuscript so does not need to be experimentally addressed.

There are a number of potential directions for future research, one in particular being combining genetic loss of hub cell quiescence (expression of Rb, activation of activin, etc) while simultaneously ablating some hub cells to address whether hub cells might be "primed" to replace themselves. However, this type of experiment is well beyond the scope of the current manuscript. Overall, this work is straightforward, clearly described and well-executed. Given the straightforward nature of the manuscript and the thoroughness of the authors in addressing the specific question under consideration, I have no significant revisions to suggest and would look forward to seeing this manuscript accepted for publication.

Reviewer 2

*Advance summary and potential significance to field*

The Drosophila testis has long served as an outstanding model for studying interactions between niches and resident stem cells. Within the Drosophila testis, a small cluster of hub cells serves as a niche for germline stem cells. A second population of cyst stem cells (CySCs) produce progeny that associate with germ cells and support their further development. Several independent groups have provided evidence that hub cells can become CySCs under certain conditions.

A single previous study from 2008 provides data that cyst stem cells and/or their progeny can also become hub cells. These results garnered widespread attention in the field. However, a follow-up study by an independent group using identical reagents failed to verify these results.

This short format study seeks to use several different orthogonal approaches to retest the ability of CySCs to give rise to new functional niche cells. First, the authors use the hub specific driver E132-gal4 in combination with GAL80ts to express the cell death inducer hid in the adult testis. Shifting flies to 31°C resulted in ablation of the hub cells and eventual loss of germ cells and somatic cells. Hub cells were not replenished during a 7-day recovery period. Additional experiments designed to result in partial ablation of the hub cells resulted in similar results: hubs generally remained small with little evidence of hub cell regeneration. Next the authors used the independent gene switch system to ablate hub cells using conditional expression of hid. Again, these experiments failed to reveal hub cell regeneration after ablation. Finally, the authors used BrdU to label cycling cells within the testis, followed by hub cell ablation. No BrdU labeling was observed in the hub under normal conditions or after hub cell ablation.
These data together support a model whereby Drosophila do not have a commonly used mechanism to replace lost hub cells.

*Comments for the author*

Overall, the paper is clearly written, and the presented experimental evidence supports the authors' conclusions. These findings will be of broad interest to the readers of Development and represents important work in the field. The experiments are well-controlled, and the given statistical analysis is appropriate and convincing. In general, I am in favor of accepting this manuscript as is, but there are a few minor points the authors could consider.

The authors state, "After one day on BrdU but before hid induction, most cells in the testis apex were labeled including GSCs, CySCs, and their progeny, as expected (Ma et al., 2014)." The authors should provide quantification of "most". Firmer conclusions could be made if all the cells outside of

© 2023. Published by The Company of Biologists under the terms of the Creative Commons Attribution License (https://creativecommons.org/licenses/by/4.0/). 

the hub were consistently labeled with BrdU before hub ablation. Perhaps extending the time of labeling could achieve this.
Alternatively, a CySC lineage trace, independent of cell division, would be even better. But I recognize that this last option would be difficult.

All the ablation experiments rely on hid. The authors could strengthen their conclusions if another gene (headcase or Dicer-1) were also examined. However, this is a minor concern.

The authors should consider including further discussion of potential reasons why their results differ from expectations based on observations presented in Voog et al (2008).

Reviewer 3

*Advance summary and potential significance to field*

The adult Drosophila testis lacks a mechanism to replenish missing niche cells by Hétié, de Cuevas, and Matunis

The hub which serves as the sustaining niche for germline and somatic (cyst) stem cells in the Drosophila testis is an important model to study niche – stem cell interactions. The hub is a defined group of usually quiescent cells. However, if cyst stem cells get lost, hub cells have the ability to become mitotically active and regenerate cyst stem cells. The cell fate change from a hub cell to a cyst stem cell is a well-established finding. What remained controversial, however, is the question whether niche cells themselves can be regenerated either by cyst stem cells or other hub cells. This is the specific problem that the authors addressed in this study. Their data strongly indicate that the hub does not regenerate after partial of full ablation.

To abolish hub cells or reduce their number and ask if they would regenerate the authors used two different methods to drive temporally limited expression of the cell death gene hid. Hub cells did not seem to regenerate in these experimental settings. The possibility that regeneration was obscured by continued elimination of hub cells during the chase period was ruled out by the findings that cell death in the hub during the chase period did not exceed that of the control, and no cycling BrdU-positive cells were observed in the hub. This revealed that remaining hub cells do not become mitotically active to replace lost neighbors and that lost hub cells are not replenished by transdifferentiating cyst cells.

Also, in this study, the authors identified a drug-inducible Gal4 driver for hub cells that might be a useful tool for further testis studies.

*Comments for the author*

The manuscript is written in a very transparent and clear manner and carefully edited. Data are demonstrated mostly by good images and are well supported by the quantitative analysis. However, there are problems with several figure panels that should be taken seriously. Please, see comments below.

Critique points:

Page 5, line 76-78: Classification of small hubs seemed to have been based not on actual quantification of size and cell number but only on estimates (‘appeared to be…’), which makes this classification subjective.
Either the hub’s width needs to be measured or the cell number. Data can be included in the text (a graph is not required).

Figure 1D: The testis seems to contain germ cells but the Vasa staining looks fuzzy and not distinct for the germ cells. Why are the Hts and DAPI stainings missing in this panel? From what I can see, this staining needs to be repeated as no proper comparison between no ablation vs ablation can be done based on this image.

© 2023. Published by The Company of Biologists under the terms of the Creative Commons Attribution License (https://creativecommons.org/licenses/by/4.0/).

Figure 1F: Page 16, lines 332-333: There is a huge variation in the sample size (from 21 to 150). Please, indicate the specific sample size for each timepoint/experiment in the graph of Fig 1F. Figure 3B: The hub still seems to be there with one cell undergoing apoptosis. But what happened to Arm, which seems to be absent between hub cells? Is the Arm staining in this image representative? If the staining is just weak in this particular image, I suggest to remove the Arm channel and outline the hub in both A and B with a stippled line, similar as done in Figure 4A-C. (The inset might not be needed here)

Figure 4C: Again, the Arm staining is not visible. In this case, the marker seems important, and the experiment might have to be repeated.

Statistics:

Page 17, line 346 and figure legend S1C state: "not significant p > 0.5". Is this correct? The cut off for significance is usually P > 0.05

Fig 2G and Fig S1C both indicate the 'error of means'. Standard deviation would be appropriate here unless the authors compare the means of several independent experiments. This is a very common mistake. As the authors show scatter blots this is not so critical, but I still suggest to correct it.

Other small points:

Figure 4 legend, line 358: replace "DAPI" with 'Arm'

Page 6, line 107: states that the normal number of hub cells is "about 16" but the graph of Fig 2G suggests that the average number is 17-18. Please, check.

Page 11, line 220, 325, and Fig. 1C: add/replace : "1B1" with 'Hts', the protein recognized by this antibody.

Page 18, line 361: remove repeated ")."

Page 18, line 364: replace "at any time point" with something like: 'after 3 (E) or 16 days (F) of recovery'

---

**First revision**

Author response to reviewers' comments

We thank the three reviewers for their insights, reproduced verbatim below in roman text. We believe we have addressed each of their concerns. Our responses are italicized.

**Reviewer 1**

Advance Summary and Potential Significance to Field:

In this work, Hetie et al have followed up on their important discovery that hub cells can exit quiescence to replenish the somatic stem cell population in the testis by addressing a critical and contentious question in the field--can somatic stem cells replenish cells of the hub. By adapting the Geneswitch system for use in the fly testis, the authors conclusively show through a number of elegant experiments that somatic stem cells do not replenish hub cells after partial or complete hub cell loss. It will be of significant interest to the field of stem cell biology to have this question resolved.

© 2023. Published by The Company of Biologists under the terms of the Creative Commons Attribution License (https://creativecommons.org/licenses/by/4.0/). 

Reviewer 1 Comments for the Author:

While use of Geneswitch as a genetic technique is novel, the results presented in this manuscript are not themselves truly novel. However, this is massively outweighed by the significant impact of these results to the field. The experiments presented are elegant and thoughtful with the results straightforward to interpret. The question of whether somatic stem cells can replace niche cells in the fly testis has been a particularly contentious issue. Experiments conducted by a lab with precisely the appropriate expertise to address this question and with experiments that provide clear-cut results is necessary for the field and as such I think warrants publication in this journal.
*We thank the reviewer for these very positive comments.*

A minor question remains as to why control testes experience hub cell loss during recovery following RU486 treatment. However, this in no way impacts the actual results/interpretation of the manuscript so does not need to be experimentally addressed.
*We think the reviewer is asking about Fig. 2G, where the mean number of hub cells in vehicle-control flies (before and after recovery / blue dots) appears slightly lower than in flies on normal food (black dots). However, as indicated on the graph, the differences are not statistically significant.*

There are a number of potential directions for future research, one in particular being combining genetic loss of hub cell quiescence (expression of Rb, activation of activin, etc) while simultaneously ablating some hub cells to address whether hub cells might be "primed" to replace themselves. However, this type of experiment is well beyond the scope of the current manuscript. Overall, this work is straightforward, clearly described and well-executed. Given the straightforward nature of the manuscript and the thoroughness of the authors in addressing the specific question under consideration, I have no significant revisions to suggest and would look forward to seeing this manuscript accepted for publication.
*Again, we thank the reviewer for positive comments and suggestions for future research.*

**Reviewer 2**

Advance Summary and Potential Significance to Field:

The Drosophila testis has long served as an outstanding model for studying interactions between niches and resident stem cells. Within the Drosophila testis, a small cluster of hub cells serves as a niche for germline stem cells. A second population of cyst stem cells (CySCs) produce progeny that associate with germ cells and support their further development. Several independent groups have provided evidence that hub cells can become CySCs under certain conditions.

A single previous study from 2008 provides data that cyst stem cells and/or their progeny can also become hub cells. These results garnered widespread attention in the field. However, a follow-up study by an independent group using identical reagents failed to verify these results.

This short format study seeks to use several different orthogonal approaches to retest the ability of CySCs to give rise to new functional niche cells. First, the authors use the hub specific driver E132-gal4 in combination with GAL80ts to express the cell death inducer hid in the adult testis. Shifting flies to 31°C resulted in ablation of the hub cells and eventual loss of germ cells and somatic cells. Hub cells were not replenished during a 7-day recovery period. Additional experiments designed to result in partial ablation of the hub cells resulted in similar results: hubs generally remained small with little evidence of hub cell regeneration. Next the authors used the independent gene switch system to ablate hub cells using conditional expression of hid. Again, these experiments failed to reveal hub cell regeneration after ablation. Finally, the authors used BrdU to label cycling cells within the testis, followed by hub cell ablation. No BrdU labeling was observed in the hub under normal conditions or after hub cell ablation. These data together support a model whereby Drosophila do not have a commonly used mechanism to replace lost hub cells.

© 2023. Published by The Company of Biologists under the terms of the Creative Commons Attribution License (https://creativecommons.org/licenses/by/4.0/).

Reviewer 2 Comments for the Author:

Overall, the paper is clearly written, and the presented experimental evidence supports the authors' conclusions. These findings will be of broad interest to the readers of Development and represents important work in the field. The experiments are well-controlled, and the given statistical analysis is appropriate and convincing. In general, I am in favor of accepting this manuscript as is, but there are a few minor points the authors could consider.
*We thank the reviewer for these very positive comments.*

The authors state, "After one day on BrdU but before hid induction, most cells in the testis apex were labeled, including GSCs, CySCs, and their progeny, as expected (Ma et al., 2014)." The authors should provide quantification of "most". Firmer conclusions could be made if all the cells outside of the hub were consistently labeled with BrdU before hub ablation. Perhaps extending the time of labeling could achieve this. Alternatively, a CySC lineage trace, independent of cell division, would be even better. But I recognize that this last option would be difficult.
*We thank the reviewer for pointing out that we were not clear in our description of this experiment and for prompting us to revisit the literature on BrdU (or EdU) labeling of testes. We had written "most cells" to refer broadly to all cells in the testis apex, some of which (eg., cyst cells) are not dividing and therefore do not incorporate BrdU. We have changed that sentence to the following: "After one day on BrdU but before* hid *induction, most cells in the testis apex were labeled, including almost all GSCs and CySCs, as expected (Yadlapalli et al., 2011; Hétié et al., 2014; Ma et al., 2014)." Our observations agree with previous reports showing that BrdU / EdU labeling of stem cells reaches a plateau at 24 hr, with 95-100% of stem cells labeled in all testes. While we agree that a CySC lineage trace experiment would be ideal, tools are not yet available for driving expression of different transgenes in CySCs vs. hub cells at the same time.*

All the ablation experiments rely on hid. The authors could strengthen their conclusions if another gene (headcase or Dicer-1) were also examined. However, this is a minor concern.
*We agree that looking at other genes would be useful, but we will reserve these experiments for the future.*

The authors should consider including further discussion of potential reasons why their results differ from expectations based on observations presented in Voog et al (2008).
*Our results agree completely with those of DiNardo et al. (2011), who were not able to reproduce results from Voog et al., and no other evidence supporting CySC conversion to hub cells has been put forward. We therefore believe the results reported in Voog et al. are an anomaly.*

**Reviewer 3**

Advance Summary and Potential Significance to Field:

The adult Drosophila testis lacks a mechanism to replenish missing niche cells by Hétié, de Cuevas, and Matunis
The hub which serves as the sustaining niche for germline and somatic (cyst) stem cells in the Drosophila testis is an important model to study niche – stem cell interactions. The hub is a defined group of usually quiescent cells. However, if cyst stem cells get lost, hub cells have the ability to become mitotically active and regenerate cyst stem cells. The cell fate change from a hub cell to a cyst stem cell is a well-established finding. What remained controversial, however, is the question whether niche cells themselves can be regenerated either by cyst stem cells or other hub cells. This is the specific problem that the authors addressed in this study. Their data strongly indicate that the hub does not regenerate after partial of full ablation.

To abolish hub cells or reduce their number and ask if they would regenerate the authors used two different methods to drive temporally limited expression of the cell death gene hid. Hub cells did not seem to regenerate in these experimental settings. The possibility that regeneration was obscured by continued elimination of hub cells during the chase period was ruled out by the findings that cell death in the hub during the chase period did not exceed that of the control, and no cycling BrdU-positive cells were observed in the hub. This revealed that remaining hub cells do not become mitotically active to replace lost neighbors and that lost hub cells are not replenished

© 2023. Published by The Company of Biologists under the terms of the Creative Commons Attribution License (https://creativecommons.org/licenses/by/4.0/).

by transdifferentiating cyst cells. Also, in this study, the authors identified a drug- inducible Gal4 driver for hub cells that might be a useful tool for further testis studies.

Reviewer 3 Comments for the Author:

The manuscript is written in a very transparent and clear manner and carefully edited. Data are demonstrated mostly by good images and are well supported by the quantitative analysis. However, there are problems with several figure panels that should be taken seriously. Please, see comments below.
*We thank the reviewer for their positive comments.*

Critique points:
Page 5, line 76-78: Classification of small hubs seemed to have been based not on actual quantification of size and cell number but only on estimates ('appeared to be…'), which makes this classification subjective. Either the hub's width needs to be measured or the cell number. Data can be included in the text (a graph is not required).
*We thank the reviewer for raising this point. Our main conclusion from this experiment is that the E132-Gal4 driver produces a wide range of outcomes, from no hub to normal hub and everything in between, and is therefore not useful for evaluating hub recovery from ablation. The precise number of testes with small vs. normal hubs does not affect this conclusion. We have therefore combined all testes that retain a hub into one category - testes with "small - normal hubs" (Fig. 1F) - and revised the text accordingly.*

Figure 1D: The testis seems to contain germ cells but the Vasa staining looks fuzzy and not distinct for the germ cells. Why are the Hts and DAPI stainings missing in this panel? From what I can see, this staining needs to be repeated as no proper comparison between no ablation vs ablation can be done based on this image.
*We thank the reviewer for pointing out that we did not provide enough explaination for what is shown in this panel. The cells that remain at the tip of this testis are older spermatocytes, which stain less brightly with Vasa, lack the distinct fusomes found in early germ cells, and have large, diffuse nuclei that do not stain well with DAPI. We have added an explanatory sentence to the figure legend: "The testis apex shown here contains only older spermatocytes, which stain less brightly with anti-Vasa."*

Figure 1F: Page 16, lines 332-333: There is a huge variation in the sample size (from 21 to 150). Please, indicate the specific sample size for each timepoint/experiment in the graph of Fig 1F.
*We have added the sample size for each timepoint / experiment to the graph.*

Figure 3B: The hub still seems to be there with one cell undergoing apoptosis. But what happened to Arm, which seems to be absent between hub cells? Is the Arm staining in this image representative? If the staining is just weak in this particular image, I suggest to remove the Arm channel and outline the hub in both A and B with a stippled line, similar as done in Figure 4A-C. (The inset might not be needed here)
*We thank the reviewer for pointing out that the Arm staining was not bright in this image. We have replaced it with another image from the same experiment, with brighter Arm staining.*

Figure 4C: Again, the Arm staining is not visible. In this case, the marker seems important, and the experiment might have to be repeated.
*Again, we have replaced the image with another with brighter Arm staining.*

Statistics:
Page 17, line 346 and figure legend S1C state: "not significant $p > 0.5$". Is this correct? The cut off for significance is usually $P > 0.05$
*Those values are indeed correct, but we have made the change as suggested by the reviewer to stick with convention.*

Fig 2G and Fig S1C both indicate the 'error of means'. Standard deviation would be appropriate here unless the authors compare the means of several independent experiments. This is a very common mistake. As the authors show scatter blots this is not so critical, but I still suggest to correct it.

© 2023. Published by The Company of Biologists under the terms of the Creative Commons Attribution License (https://creativecommons.org/licenses/by/4.0/).

*We thank the reviewer for this comment. We have revised the graphs to show mean and standard deviation.*

Other small points:
Figure 4 legend, line 358: replace “DAPI” with ‘Arm’
*We thank the reviewer for catching this error. We have corrected it.*
Page 6, line 107: states that the normal number of hub cells is “about 16” but the graph of Fig 2G suggests that the average number is 17-18. Please, check.
*As shown in Fig. 2G,* GS-2295-Gal4>hid *flies have an average of 17-18 hub cells before induction vs. about 16 after a mock (vehicle only) induction, but there is no significant difference between the two. Both averages are slightly higher than numbers reported for true wild type flies: 10-15 hub cells/testis (reviewed in Greenspan et al., 2015). We have added this information to the Introduction.*

Page 11, line 220, 325, and Fig. 1C: add/replace : “1B1” with ‘Hts’, the protein recognized by this antibody.
*The* hts *gene encodes several distinct proteins that are located in different structures and recognized by different antibodies (Petrella et al., 2007, Development). To avoid confusion with other "Hts" antibodies, we prefer to use "1B1", which is standard practice in our field.*

Page 18, line 361: remove repeated “).”
*We thank the reviewer for catching this typo. We have corrected it.*

Page 18, line 364: replace “at any time point” with something like: ‘after 3 (E) or 16 days (F) of recovery’
*We thank the reviewer for pointing out that this sentence (which refers to both experiments) was not clear. We have replaced it with this: "Proliferating germline and somatic cells* outside *the hub are robustly labeled with BrdU initially (A, D), but we found no BrdU-labeled cells* inside *the hub in any testis at any time point in either experiment (A-F)."*

*In addition to the changes outlined above, we have also condensed the Abstract to comply with the word limit for short reports and updated the Methods to include catalog numbers for antibodies.*

---

Second decision letter

MS ID#: DEVELOP/2022/201148

MS TITLE: The adult Drosophila testis lacks a mechanism to replenish missing niche cells

AUTHORS: Phylis Hétié, Margaret de Cuevas, and Erika L Matunis
ARTICLE TYPE: Research Report

I am happy to tell you that your manuscript has been accepted for publication in Development, pending our standard ethics checks.

Reviewer 2

*Advance summary and potential significance to field*

*Comments for the author*

The authors have addressed all of my previous concerns.

I find the paper acceptable for publication.

© 2023. Published by The Company of Biologists under the terms of the Creative Commons Attribution License (https://creativecommons.org/licenses/by/4.0/).

Reviewer 3

*Advance summary and potential significance to field*

see review of first manuscript version

*Comments for the author*

My comments and suggestions have been addressed. The Arm stainings of the hub are now excellent.

© 2023. Published by The Company of Biologists under the terms of the Creative Commons Attribution License (https://creativecommons.org/licenses/by/4.0/).
